# Mode of allocation and social demographic factors correlate with impaired quality of life after liver transplantation

**DOI:** 10.1186/s12955-015-0360-z

**Published:** 2015-09-30

**Authors:** Nils Heits, Gunnar Meer, Alexander Bernsmeier, Rainer Guenther, Bjoern Malchow, Thomas Kuechler, Thomas Becker, Felix Braun

**Affiliations:** Department of General, Visceral-, Thoracic-, Transplantation- and Pediatric Surgery, University Medical Centre Schleswig-Holstein (UKSH), Campus Kiel, Arnold-Heller Strasse 3 (Haus 18), 24105 Kiel, Germany; Institute of Department of Internal Medicine I, University Medical Centre Schleswig-Holstein (UKSH), Campus Kiel, Arnold-Heller Strasse 3 (Haus 18), 24105 Kiel, Germany; Reference Center for Quality of Life, University Medical Centre Schleswig-Holstein (UKSH), Campus Kiel, Arnold-Heller Strasse 3 (Haus 18), 24105 Kiel, Germany

**Keywords:** Liver transplantation, Quality of life, Allocation-system, Social demographic factors, Age, Sex, HCC, Waiting time

## Abstract

**Background:**

Health-related Quality of life (HRQoL) is a major goal of clinical management after liver transplantation (LTx). There is still disagreement on the effects of social-demographic factors and changes in the allocation system on HRQoL. The aim of this study was to evaluate the impact of social-demographic factors, mode of organ-allocation, waiting time and hepatocellular carcinoma (HCC) on HRQoL after LTx.

**Methods:**

HRQoL was assessed using the EORTC-QLQ-C30 questionnaire, which was sent to 238 recipients. Investigated parameters included age, sex, distance to transplant center, follow-up at hospital, size of hometown, highest education, marital status, having children, background liver disease, waiting time, mode of allocation, HCC, hospitalization after LTx and diagnosis of malignancy after LTx. All evaluated parameters were entered into multivariate linear regression analysis.

**Results:**

Completed questionnaire were returned by 73 % of the recipients. After LTx, the HRQoL-function scales increased over time. Age, marital status, highest education, completed professional training, working status, job position, duration of waiting time to LTx, distance to transplant center, place offollow, HU-statuts, mode of organ allocation and duration of hospitalization were associated with significantly worse function- and significantly lower symptom scales. HCC as a primary disease did not affect HRQoL.

**Conclusions:**

Low HRQoL correlated significantly with MELD-based organ allocation, more than 28-day hospitalization, divorced status, lower education- and non-working status, higher distance to transplant center, follow up at transplant center, HU-status, shorter waiting time to LTx and younger age. Improvement of HRQoL after LTx may require clinical management of pain, psychotherapy and financial support.

## Introduction

Since the reporting of the first liver transplantation (LTx) in 1963 by Starzl et al. [1], the estimated overall 1-year and 5-year survival rates exceed 85 and 70 % [2] and LTx has become the standard therapy for end-stage liver disease. In the past years, health-related quality of life (HRQoL) after LTx has gained more interest in the transplantation community. HRQoL is assessed by a questionnaire that encompasses physical ability, psyche, socio-economic factors and interpersonal level measured over certain period of time. Several studies reported homogeneous results concerning HRQoL after LTx and showed a significant improvement of physical efficiency one year after LTx [3–5]. Such improvement covered fatigue, weakness and coping with problems of everyday life activities [6–8]. Especially, recurrence of liver disease [8–11] and ability to work were identified as main factors to influence HRQoL after LTx. However, there is still disagreement on whether HRQoL is influenced by various social demographic factors.

The aim of this study was to identify social demographic factors that influence HRQoL after LTx. We focused on the effects of age at the time of LTx, job position as well as education- and working-status, since LTx is being conducted increasingly in older patients over the years and financial difficulty has been identified to be a major problem after LTx. We also evaluated the effects of marital status and social networking, since LTx patients might benefit from the help of family members or friends after transplantation. We also evaluated the effects of the new mode of organ allocation. Specifically, the European Liver Allocation System (ELAS) was replaced in 2006 by the Model for End-Stage Liver Disease (MELD) to reduce mortality on the waiting list and to optimize organ allocation for LTx. Based on this change, hepatocellular carcinoma (HCC) became the second most common indication for LTx and patients with HCC could undergo LTx through a faster allocation using the implemented match-MELD, where patients with stable HCC disease inside the Milan Criteria (MC) are transplanted by primary donor organs. The stable tumor disease within MC needs to be verified every 3 months and leads to additional match-MELD-points corresponding to +10 % of the 3-month mortality. Based on the increased proportion of patients with HCC on the LTx waiting list, we analyzed the impact of HCC, compared to non-tumor liver disease, on HRQoL. In addition, we analyzed the impact of the waiting time to LTx on the HRQoL.

## Material and methods

### Study cohort

Between 1988 and 2013, 660 adult patients underwent LTx at the UKSH, Campus Kiel. Of these 660, 252 patients fulfilled the criteria used for study entry and were enrolled in the study. The inclusion criteria were 1) patients who had undergone a deceased donor organ LTx, and 2) patients who underwent re-transplantation for organ failure. The exclusion criteria were 1) Patients younger than 16 years, 2) patients who had died during follow-up, 3) patients with a combination organ transplantation, 4) living donor liver transplant recipients, and 5) non-residents. Patients were followed up for a median of 172 months (8–769 months). Each patient was followed-up in the Outpatient Clinic or at the office of a practicing physician at 3, 6 and 12 months after LTx, and also every 12 months for those patients who had uneventful course.

### Study design

After approval of the study by the local ethics committee, a self-administered EORTC QLQ-C30-questionnaire with an additional LTx questionnaire to assess the HRQoL was sent to selected patients for cross-section analysis. Each questionnaire sheet was sent with an informed consent sheet; the latter was signed by the patient. The questionnaire contained 30 main questions related to various diseases. The EORTC QLQ-C30-questionnaire has 5 function scales (physical functioning, role functioning, cognitive functioning, emotional functioning, social functioning) and 3 symptom scales (fatigue, pain, nausea, vomiting). Moreover, the questionnaire included a Global Health-scale, Quality of Life-scale and additional parameters commonly used to assess typical clinical symptoms/signs and status of cancer patients (dyspnea, loss of appetite, sleep disturbance, constipation, diarrhea, financial difficulties) [12]. These scales were supplemented by social demographic data, including age, sex, marital status, school and professional education, number of children, size of hometown, working status and working position [12]. For easier interpretation, the assessed scores were transformed to a scale ranging from 0 to 100. Thus, high levels of global health, QOL and function scales indicated a higher ability, whereas high scores of symptom-scales indicated suffering of the patient [12].

The following eleven social demographic parameters were entered in the correlation analysis: age at time of LTx (16 to <47 years vs. ≥47 to <57 years vs. ≥57 years), sex, marital status, presence of children, highest graduation, completed professional training, working status (employed, unemployed), job position, size of hometown, place of follow up after LTx and distance between home and transplant center (≤155 km vs. >155 km). A cutoff value of 155 km was chosen as it reflects the distance to the next transplant center. Furthermore, the following six allocation parameters were entered in the correlation analysis: urgency for LTx (HU for high urgent indication, T for patients with chronic disease), duration of waiting before LTx (<180 days vs. ≥180 days), era of organ allocation (ELAS- vs. MELD-era), presence or absence of HCC, duration of hospitalization at transplant center (<28 days vs. ≥28 days), and presence or lack of malignant tumor after LTx. A cutoff of 180 days waiting before LTx was chosen as patients transplanted after less than 180 days were in most cases more instable with a higher priority than patients who waited longer. The cutoff of 28 days patient hospitalization was chosen as most patients with a hospital stay longer than 28 days after transplantation were hospitalized due to complications after surgery.

To quantify improvement in function- and symptom-scores, we compared the scores and QOL-assessment before LTx to those at follow-up. For meaningful comparison, the study cohort was divided into three groups; the 0 to <53 group (representing those who underwent LTx 0 to <53 months before assessment), the ≥53 to <114 group (≥53 to <114 months) and the ≥114 group (≥114 months). Cutoffs between the groups of age at time of LTx were chosen to reflect different stages of social and working life. As a control group, we used the reference data of the common German society, which were also evaluated by an EORTC QLQ-C30-questionnaire by Schwarz and Hinz [13].

### Statistical analysis

All metric parameters were expressed as total numbers (%) or mean ± standard deviation. To test the influence of social demographic and clinical parameters on Quality of Life-Functioning and -Symptom Scores we performed a simple and multiple linear regression analysis. Significant variables in unviariate analysis were then included in stepwise forward multiple linear regression to perform multivariate testing. Positive values for stadardized regression coefficient b lead to an increase, negative values to a decrease in tested functioning and symptom score items. Collinearity was not present with tolerance values well above 0.2. A *p* value <0.05 was considered statistically significant. All analyses described in this paper were conducted using the Statistical Package for Social Sciences software (version 22.0, SPSS Inc., Chicago, IL).

## Results

### Study population

At the time of the QOL-assessment, the questionnaire was sent to 238 patients. Of the 252 patients, 14 patients had transferred and followed up at a different transplant center and therefore their data were excluded from the analysis. Of the remaining 238 patients, completed questionnaires were returned by 173 patients (73 %). The reason for the no return of the questionnaire in the remaining 65 patients was unwillingness to return in 39 (15.5 %) and 26 (10.3 %) had died or moved to foreign countries after surgery. Among the 173 patients that returned the questionnaire, 19 (7.5 %) were relisted for LTx. The mean age of the patients at the time of LTx was 52.7 ± 11.9 years. Most of the patients were ≥57 years old (40 %), followed by the group ≥47 to <57 (33 %) and the group <47 years (27 %) (Table 1). Of these patients 117 (49.2 %) underwent LTx during the ELAS-era and 121 (50.8 %) during the MELD-era. The mean waiting time till LTx was 317.6 ± 484.8 days. During the ELAS-era, the mean waiting time was 336 days and the mean MELD-score was 15. In the MELD-era, the waiting time was shorter (302 days) and MELD-score was higher (19 points). The mean duration of hospitalization was longer for patients in the ELAS-era (36 days), compared with that in the MELD-era (32 days). Only 18 (7.8 %) of the patients required urgent transplantation. The background liver diseases were alcoholic cirrhosis 53 (31 %), hepatitis C-cirrhosis in 22 (13 %), HCC in 21 (12 %), cholestatic liver disease in 20 (12 %), cryptogenic cirrhosis in 10 (6 %), benign liver tumors and polycystic liver diseases in 11 (6 %), acute hepatic failure in 9 (5 %), hepatitis B in 8 (5 %), autoimmune hepatitis in 8 (5 %), metabolic liver disease in 9 (5 %), Budd-Chiari-syndrome in 1 (1 %) and non-gastrointestinal secondary liver tumors in 1 (1 %) patient.

**Table 1:** Assessed social demographic factors using the EORTC QLQ-C30-questionnaire

Parameter	n (%)
Sex	
Males	*n* = 99 (59 %)
Females	*n* = 69 (41 %)
Age (years)	
16 to <47 years	*n* = 46 (27 %)
≥ 47 to <57 years	*n* = 58 (33 %)
≥ 57 years	*n* = 69 (40 %)
Marital status	
Single	*n* = 21 (13 %)
Married	*n* = 115 (70 %)
Relationship	*n* = 7 (4 %)
Divorced	*n* = 16 (10 %)
Widowed	*n* = 7 (4 %)
Children	
Yes	*n* = 123 (74 %)
No	*n* = 43 (26 %)
Living alone	
Yes	*n* = 35 (26 %)
No	*n* = 99 (74 %)
Size of hometown	
Major city (population >100,000)	*n* = 41 (25 %)
Town (population 50,000–100,000)	*n* = 16 (10 %)
Small town (population 5000–50,000)	*n* = 52 (32 %)
Village (population–5000)	*n* = 54 (33 %)
Highest education	
Secondary school certificate	*n* = 67 (40 %)
Technical college qualification	*n* = 17 (10 %)
No graduation	*n* = 3 (2 %)
Secondary modern school certificate	*n* = 58 (35 %)
University-entrance diploma	*n* = 22 (13 %)
Completed professional training	
Traineeship	*n* = 97 (69 %)
Technical college	*n* = 18 (13 %)
Advanced technical college	*n* = 8 (6 %)
University	*n* = 7 (5 %)
No education	*n* = 10 (7 %)
Employment status	
Working	*n* = 26 (17 %)
Housewife/husband	*n* = 9 (6 %)
Retired	*n* = 105 (68 %)
Certified unfit for work	*n* = 5 (3 %)
Unemployed	*n* = 5 (3 %)
Other	*n* = 5 (3 %)
Job position	
Worker	*n* = 18 (18 %)
Employee	*n* = 42 (42 %)
Official	*n* = 12 (12 %)
Executive employee	*n* = 89 (9 %)
Self-employed	*n* = 12 (12 %)
Other	*n* = 7 (7 %)

Most patients were followed-up at the Outpatients Clinic of our transplantation center, and only a small number of patients (*n* = 23, 13 %) were followed up at private clinics.

### Social demographic characteristics

The study cohort comprised 99 males and 69 females (Table 1), and the majority were married (69.5 %). Furthermore, a large proportion lived at least with one other person; and 74 % had children. Most patients lived in villages or small towns. With regard to the level of highest education, the largest group had secondary modern school, followed by a secondary school certificate, university-entrance diploma and technical college qualification, and only 2.4 % had no graduation. The largest group completed traineeship, followed by technical college, advanced technical college or university degree (Table 1), whereas 7 % did not state the professional education. With regard to job position, the majority of patients were retired, followed by current workers, unemployed and confirmed unfit for work (Table 1). Among the working patients, the largest proportion were employees, followed by workers, officials, self-employed and leading executives (Table 1).

### HRQoL-characteristics

The mean score was 71.6 for physical functioning, 64 for role functioning and 67.5 for emotional functioning. Furthermore, the mean scores for cognitive functioning, social functioning and global health status were 73.1, 67.9 and 65.4, respectively. The mean scores for the symptom-score-assessment were 41.6 for fatigue, 9 for nausea and vomiting, 32.6 for pain and 27.8 for dyspnea. For insomnia, the mean score was 39.3, for appetite loss 14.1, for constipation 12.9, for diarrhea 17.6 and 34.5 for financial difficulties. Comparison of our data with those of Schwarz and Hinz (13), who measured HRQoL in 2001 people of common German society using the EORTC QLQ-C30-questionnaire, showed clinically relevant worse HRQoL-scores in our study for fatigue, pain, dyspnea, insomnia, diarrhea and financial difficulties. However, the HRQoL-scores for nausea/vomiting, appetite loss, global health status and constipation were comparable to those reported in the above study.

**Fig. 1:**
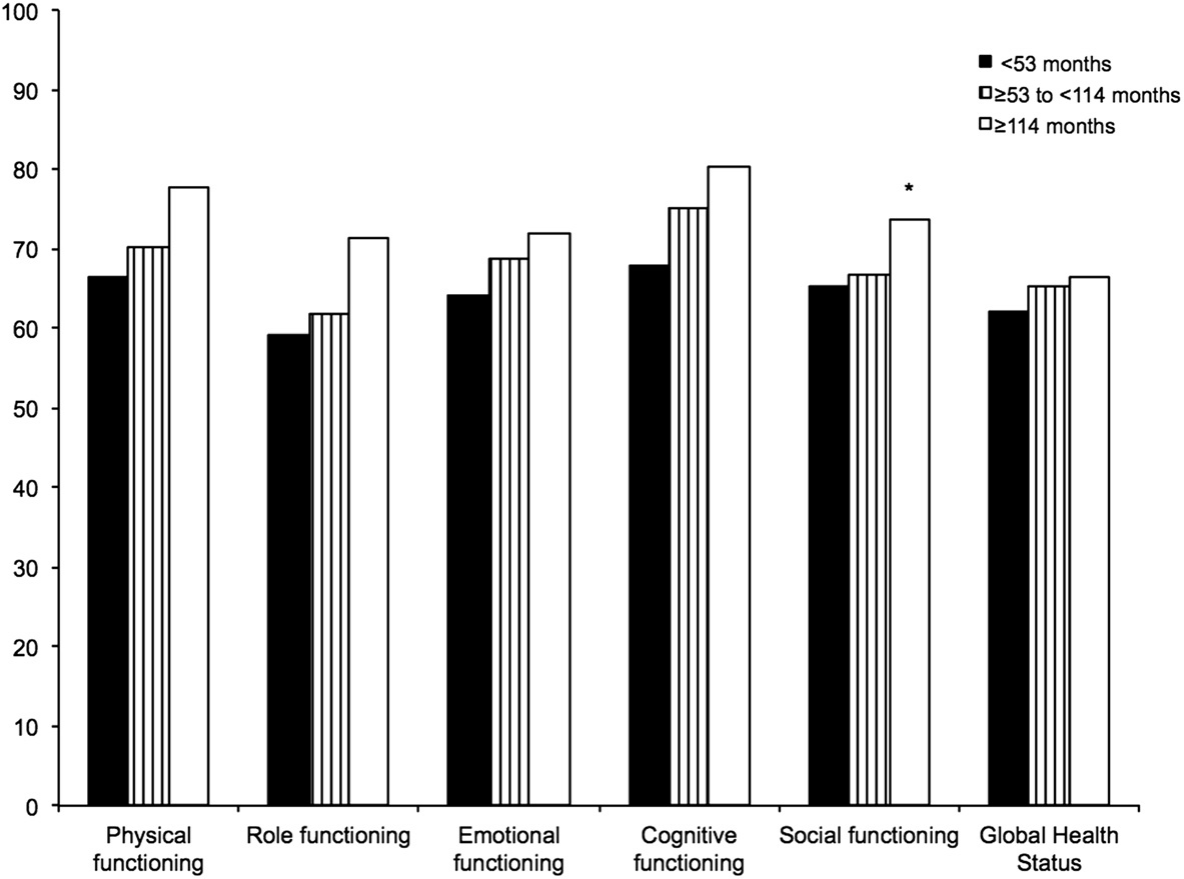
Correlation of functioning scores to time between LTx and HRQoL-assessment (*p < 0.05)

Further analysis of the functioning scores showed expected improvement in all functioning scores with longer follow-up period (between LTx and the HRQoL-assessment). The social functioning scale was significantly different among the groups (*p* = 0.013) (Fig. 1). The symptom scores tended to improve at ≥114 months after LTx, but the difference for any of the assessed symptoms was not statistically significant.

### Results of correlation of social-demographic parameters after multivariate testing

Correlating the age of the patients at the time of LTx, we measured less constipation in the <47 patients (*p* = 0.032) and less financial difficulties for patients >57 years (*p* = 0.003). The mean score for emotional functioning increased with aging. In contrast, financial difficulties were more common among younger age patients than older patients. With regard to gender differences in HRQoL, larger proportion of women suffered fatigue and insomnia than men. Furthermore, cognitive functioning tended to be better in women than men albeit insignificantly.

**Fig. 2:**
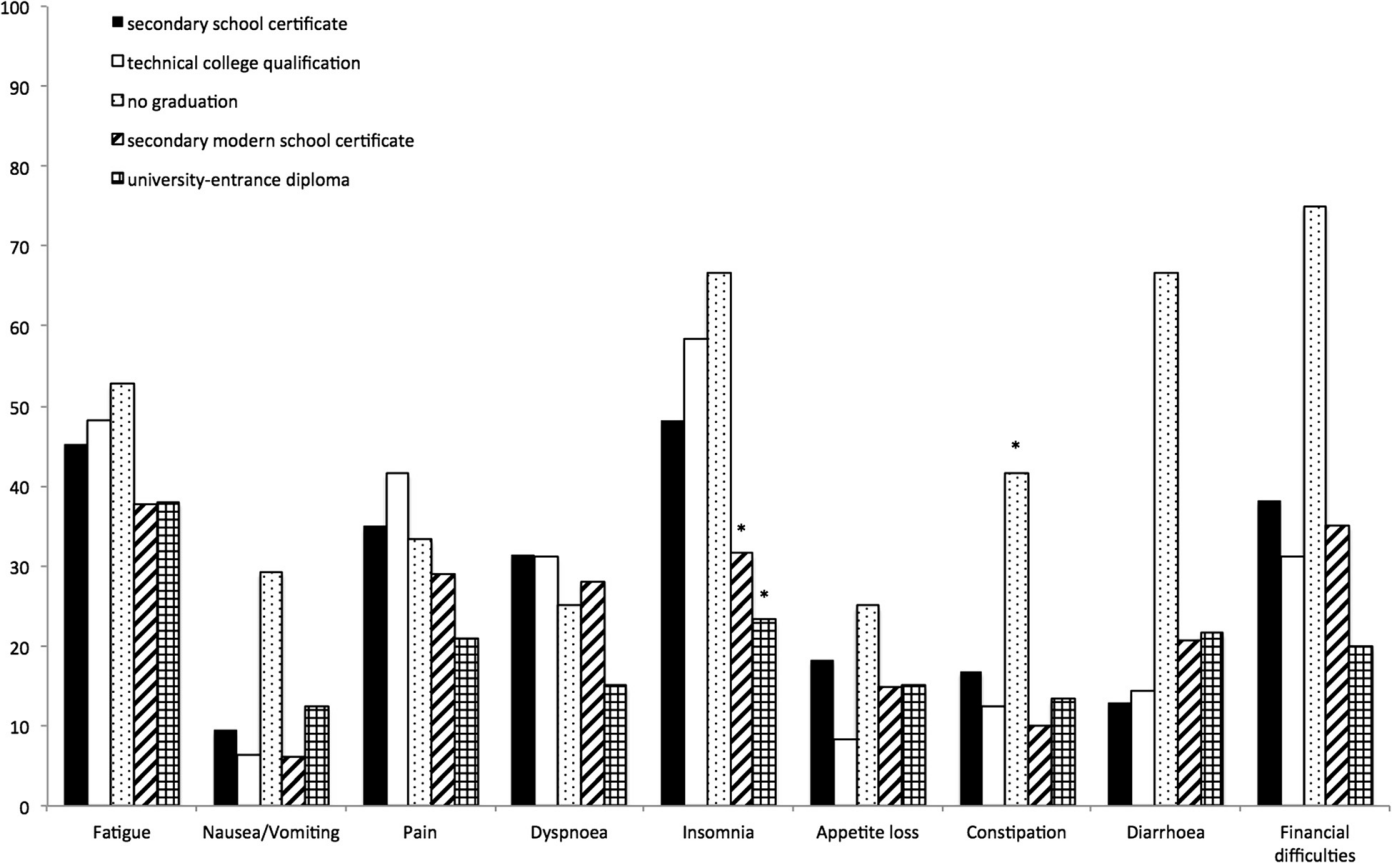
Correlation of symptom scores to highest education (*p < 0.05)

**Fig. 3:**
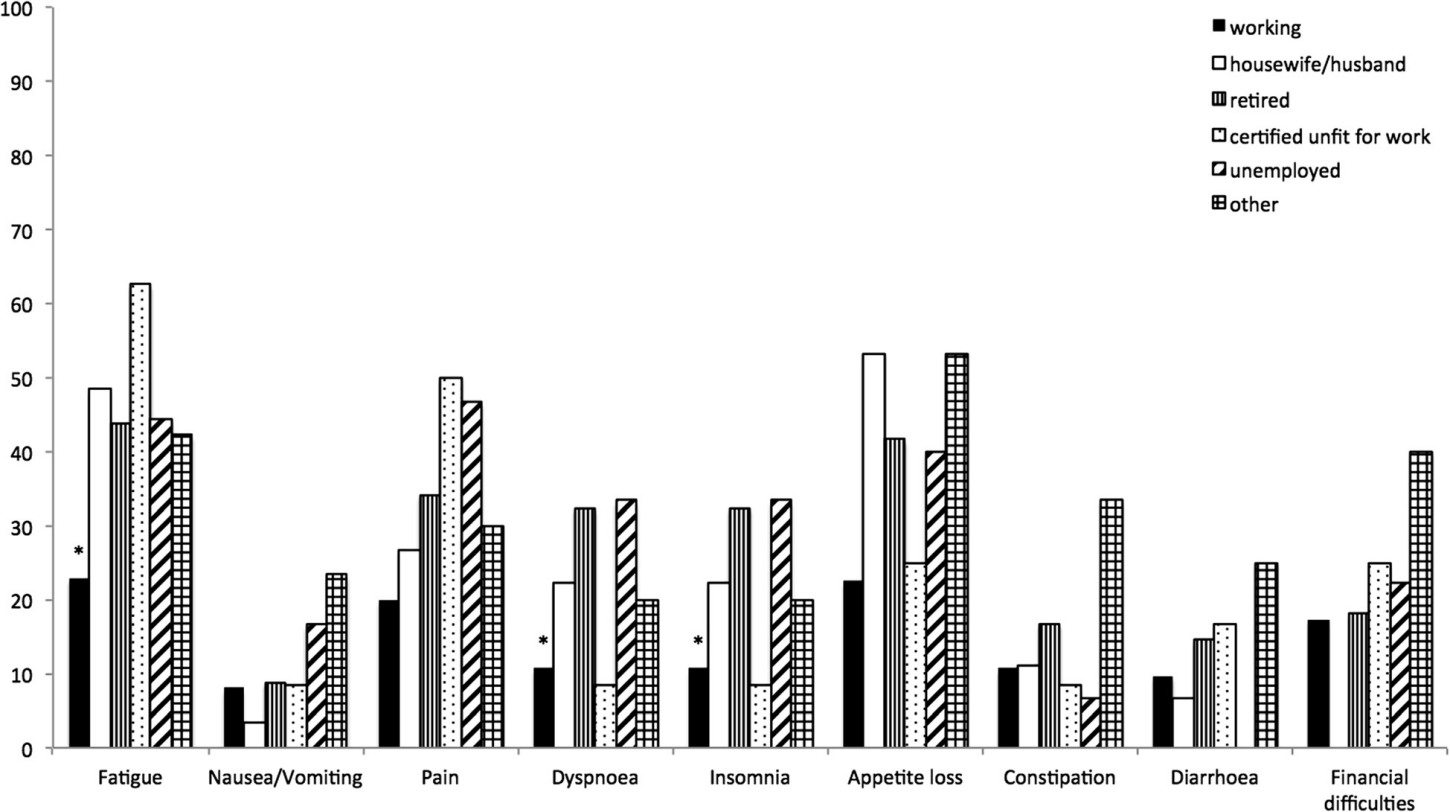
Correlation of symptom scores to employment status (*p < 0.05)

**Fig. 4:**
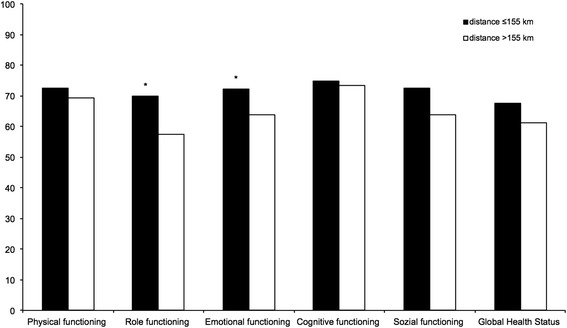
Correlation of functioning scores to distance to transplant center (*p < 0.05)

We also assessed the role of family and social network by examining the effects of marital status on HRQoL. HRQoL-scores for divorced patients were significantly worst in physical functioning (*p* = 0.004). Surprisingly, single patients, widowed patients, patients living alone and having children had no effect on HRQoL. Further analysis showed significantly worse HRQoL-scores for patients with a lower education and graduation. Patients with a job had significantly better HRQoL-scores. Patients with no graduation had significantly worst HRQoL-scores for cognitive functioning (*p* = 0.03) and constipation (*p* = 0.022). Patients with secondary school certificate had significantly worst functioning scores for global health status (*p* = 0007). Patients with technical college degree (insomnia: *p* = 0.002), secondary modern school certificate (financial difficulties: *p* = 0.032) and university-entrance diploma (insomnia: *p* = 0.009) showed significantly better symptom scores for insomnia and financial difficulties (Fig. 2). An unexpected significant worse symptom score for diarrhea was measured in patients with technical college degree (*p* = 0.012). Patients with no education had significantly worst symptom-scores for diarrhea (*p* = 0.047) and financial difficulties (*p* = 0.042). Workers had worse symptom-scores in nausea and vomiting (*p* = 0.005). Being employed was correlated with better HRQoL-scores. Working patients had significantly better scores in physical functioning (*p* = <0.001), role functioning (*p* = 0.01), global health status (*p* = 0.02), fatigue (*p* = 0.002), dyspnoe (*p* = 0.008) and insomnia (*p* = 0.032) (Fig. 3). To test the influence of a high distance, that patients had to travel for their follow up examinations, we compared HRQoL-scores for patients with long and short distance to the transplant center. Living in a distance >155 km was associated with significantly worst HRQoL-scores for role functioning (*p* = 0.023), emotional functioning (*p* = 0.035), fatigue (*p* = 0.014) and dyspnoe (*p* = 0.046) (Fig. 4). Follow-up at transplant center had worst HRQoL-scores for emotional functioning (*p* = 0.012), cognitive functioning (*p* = 0.009), fatigue (*p* = 0.022) and insomnia (*p* = 0.038). Size of hometown had no effect on HRQoL. All significant HRQoL-scores are shown in Table 2.

**Table 2:** Significant symptom- and functioning-scores related to social demographic parameters

Correlated parameter	Mean value (±SD)	*p*-value univariate	*p*-value multivariate
Working:			
● Physical Functioning	92 (±11,1)	<0.001	<0.001
● Role Functioning	82 (±22.5)	0.001	0.01
● Global Health Status	77 (±24.1)	0.005	0.02
● Fatigue	23 (±20.1)	<0.001	0.002
● Dyspnoe	11 (±20.9)	0.004	0.008
● Insomnia	23 (±31.5)	0.013	0.032
● Pain	20 (±28.1)	0.035	n.s.
Retired:			
● Physical Functioning	66 (±23.4)	0.001	n.s.
● Role Functioning	59 (±32.6)	0.038	n.s.
● Dyspnoe	32 (±33.6)	0.003	n.s.
No graduation:			
● Cognitive Functioning	46 (±25)	0.027	0.03
● Constipation	25 (±31.9)	0.024	0.022
● Financial Difficulties	75 (±16.7)	0.034	n.s.
Secondary school:			
● Global Health Status	68 (±23.6)	0.013	0.007
● Insomnia	48 (±36.6)	0.031	n.s.
● Diarrhea	13 (±20.9)	0.04	n.s.
Secondary modern school:			
● Cognitive Functioning	79 (±24.1)	0.033	n.s.
● Insomnia	32 (±35.6)	0.017	0.002
Technical college qualification:			
● Insomnia	58 (±37.5)	0.045	n.s.
University-entrance diploma:			
● Pain	21 (±30.5)	0.046	n.s.
● Insomnia	23 (±24.4)	0.021	0.009
No education:			
● Diarrhea	20 (±35.8)	<0.001	<0.001
● Financial Difficulties	63 (±33.1)	0.011	0.042
Traineeship:			
● Dyspnoe	30 (±32.7)	0.038	n.s.
Technical college:			
● Diarrhea	20 (±28.1)	0.034	0.012
● Financial Difficulties	31 (±37.5)	0.041	0.032
Age <47 year:			
● Role Functioning	74 (±27.7)	0.01	n.s.
● Physical Functioning	81 (±18.5)	0.001	n.s.
● Constipation	7 (±15.5)	0.032	0.032
● Financial Difficulties	47 (±40.5)	0.007	n.s.
Age ≥57 years:			
● Physical Functioning	65 (±22.9)	0.003	n.s.
● Diarrhea	11 (±20.8)	0.018	n.s.
● Financial Difficulties	22 (±33.2)	<0.001	0.003
Divorced:			
● Physical Functioning	71 (±25.1)	0.039	0.04
Living alone:			
● Pain	18 (±29.4)	0.029	n.s.
Married:			
● Diarrhea	15 (±24.2)	0.05	n.s.
Distance to transplant center >155 km:			
● Role Functioning	58 (±33.7)	0.011	0.023
● Emotional Functioning	64 (±26.1)	0.031	0.035
● Fatigue	47 (±29.4)	0.017	0.014
● Dyspnoe	34 (±33.3)	0.01	0.046
Follow up at transplant center:			
● Emotional Functioning	66 (±25.5)	0.01	0.012
● Cognitive Functioning	72 (±25.3)	0.006	0.009
● Fatigue	44 (±27.8)	0.005	0.022
● Insomnia	43 (±36.3)	0.028	0.038
Size of hometown (population 50.000–100.000):			
● Global Health Status	53 (±22)	0.034	n.s.

**Table 3:** Significant symptom- and functioning-scores related to allocation-related parameters

Correlated parameter	Mean value (±SD)	*p*-value univariate	*p*-value multivariate
Duration of waiting before LTx ≥180 days:			
● Emotional Functioning	72 (±21.6)	0.036	n.s.
● Fatigue	36 (±25.9)	0.009	0.023
● Nausea/Vomiting	5 (±14.7)	0.012	n.s.
● Apetite loss	10 (±23.6)	0.012	–
Hospital stay ≥28 days:			
● Physical functioning:	67 (±23.3)	0.021	0.003
● Role functioning:	58 (±30.7)	0.022	0.05
● Emotional functioning:	63 (±26.3)	0.005	0.007
● Social Functioning:	61 (±34.2)	0.006	–
● Fatigue	46 (±7.32)	0.037	n.s.
● Dyspnoe	33 (±33.5)	0.026	n.s.
●Constipation	9 (±17.5)	0.036	n.s.
● Financial Difficulties	41 (±40.5)	0.025	n.s.
Presence of HCC:			
● Physical Functioning	64 (±26.1)	0.02	n.s.
Organ allocation in ELAS-era:			
● Physical Functioning	75 (±22.5)	0.022	0.001
HU-status:			
● Financial Difficulties	61 (±41.7)	0.017	0.025

### Results of correlations of allocation-related parameters after multivariate testing

**Fig. 5:**
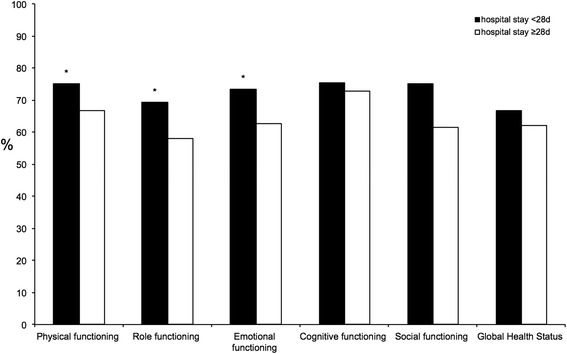
Correlation of functioning scores to duration of hospitalization (*p < 0.05)

Among patients who underwent LTx after more than 180-day waiting period, the HRQoL-score was significantly better for fatigue (*p* = 0.023). The HRQoL-scores for physical functioning (*p* = 0.003), role functioning (*p* = 0.05) and emotional functioning (*p* = 0.007) were significantly worse in patients with a hospital stay of ≥28 days (Fig. 5). A significantly higher score for physical functioning (*p* = 0.001) was noted in patients transplanted before the MELD-allocation era. HU-status at time of transplantation was associated with a significantly higher symptom score for financial difficulties. There was no significant difference in HRQoL based on background liver disease. Furthermore, there was no significant difference in the incidence of malignant tumor after LTx. All significant HRQoL-scores are shown in Table 3.

## Discussion

The present study assessed the impact of social-demographic factors and changes in the allocation system on HRQoL, since the influence of these factors remain controversial.

Using available data on German society as reference, we assessed relevant low HRQoL for patients who underwent LTx. The results were comparable to those published by other investigators [6, 8, 14, 15]. One surprising result was the global health status of the study patients, which was only slightly worse in LTx recipients. One possible reason for this observation is the structure of the EORTC QLQ-C30-questionnaire whereas the last two questions serve as subjective assessment of potential happiness. Happiness might be stronger among patients that survive LTx compared to healthy people, who cannot express the feelings of surviving severe disease in the same way as patients who undergo LTx. Thus, we speculate that healthy people tend to rate these questions less positively.

We measured an improvement of HRQoL with longer period between LTx and HRQoL-assessment. This finding could be due to the fact that patients who are too sick to fill out the questionnaire or those who have died are not included in the assessment of the impact of this factor. Nevertheless, our results are similar to those of other groups who reported improvement in QOL at one year after LTx [3, 6, 8–11, 14, 15]. The reported effect of age on HRQoL is controversial. For example, Desai et al. [15] reported significantly worse HRQoL for patients aged ≥50 years at the time of LTx, while Saab et al. [8] indicated that the effect was marginal and not significant in their patients aged ≥60 years, and Aberg et al. [14] concluded that old age did not affect HRQoL. Our analysis showed that young age (16-47 years) correlated significantly with better constipation and a trend of more financial difficulties, as this score was only significantly worst in the univariate analysis. Correspondingly, old patients (>57 years) had a significantly lower score for financial difficulties. This finding might be related to the better financial security or early retirement of older recipients, better social integration and perhaps better family background. The notion of better HRQoL associated with financial security, higher social status and social background was confirmed by the finding of significantly worse physical functioning in divorced patients, which might reflect depression after LTx. Furthermore, better HRQoL-scores were also noted in patients with higher education, higher graduation and employed patients, with associated higher income and probably high social status. These results are similar to those of earlier studies, which showed that employment or earlier return to employment after LTx was associated with better HRQOL [14, 16, 17], and conversely, that low monthly family income per capita was associated with low HRQoL-scores [18]. Our finding of the lack of relationship between gender and HRQoL is similar to that of previous studies [8, 14], but different from others [9, 15] who showed poor HRQoL-scores in LTx women. Further studies are needed to establish the effect of gender on the HRQoL score.

Of special interest was the finding related to the effect of distance from the transplant center. This became especially important since 2007 after noticing a higher number of concerned patients who lived far away from the transplant center. These concerns are potentially related to the withdrawal of payments costs of travel from home to the transplant center by the German health insurance in 2007. This point is peculiar to Germany and is especially important where reduction of transplant centers is currently being discussed by the German health care system. Any such reduction would probably result in further financial burden to the patients, with potential low compliance and lesser visits to the medical care centers for post LTx-follow up. This might worsen HRQoL for such patients. In this study, higher distance to transplant center correlated with significantly lower HRQoL. However, financial difficulties did not correlate with a higher distance to the transplant center. Financial difficulties were significantly higher in younger patients, divorced patients, low educated, unemployed patients or patients with HU-status after transplantation. For HU-status patients the sudden event of liver failure is a dramatic event, where patients are pulled out of their every day life and working position. Therefore, financial support should be provided to this group of patients to compensate for higher travel costs. The significantly worst HRQoL-scores for patients followed up at the transplant center were surprising. An explanation could be, that most patients followed up at our transplant center are patients with complications after transplantation. Another explanation could be a negatively influence on HRQoL-scores in patients who filled out the questionnaire after a longer journey to the transplant center’s outpatient clinic.

The MELD-allocation system was established in the Eurotransplant area in 2006. While the system reduced waiting-list mortality, it also reduced the one-year survival rate after LTx [19]. The reported correlation between HRQoL and pre-transplant MELD-score varies widely among the published studies, where authors indicated that high pre-transplant MELD-scores correlate with higher mortality rate after LTx [20], whereas others [11, 21] report better physical functioning in patients with high MELD-scores compared to those with low MELD-scores. Saab et al. [22] concluded that pre-transplant MELD-score did not influence post-LTx HRQoL. To our knowledge, there are no studies that compared the effects of different allocation-systems on post-LTx HRQoL. Our results showed significant effect for the allocation-era on post-LTx HRQoL. Physical functioning was significantly better in patients who received LTx during the ELAS-era than the MELD-era. In this context, patients with shorter follow-up had overall worse HRQoL-scores compared with longer follow-up period. On the other hand, patients who underwent LTx during the ELAS-era had a lower pre-transplant MELD-score than the MELD-era. Furthermore, the waiting time was longer in patients who received LTx during the ELAS-era. The fact that patients with longer waiting time had significantly better symptom-scores than those with shorter waiting time suggests that patients who underwent LTx during the ELAS-era were less sick than those during the MELD-era. The duration of hospitalization was longer for patients who underwent LTx during the ELAS-era. This finding is probably related to the improvement in postoperative care and medical system at our center after 1988. It should be noted that a significant proportion of patients who received LTx during the MELD-era were transferred after hospital discharge to rehabilitation centers, which could have distorted the data on duration of hospitalization during the MELD-era. Nevertheless, patients with mean hospital stay ≥28 days had significantly worse HRQoL for most of the evaluated functioning-scores.

Our results showed no significant difference in HRQoL between patients with and without HCC patients. The same finding was noted in patients of the long-term follow-up group who were diagnosed with malignancy after LTx. This might be due to the differences in the number of patients with and without HCC and the small number of patients who developed malignancies after LTx. In this regard, there is little or no information on HRQoL of HCC-patients after LTx. One study by Mabrouk et al. [23] reported significantly lower HRQoL in transplanted HCC-patients. Furthermore, another study showed a significant effect on the composite mental HRQoL score, but did not further specify the neoplasms entity [11]. Aberg et al. [14] also showed no significant difference in HRQoL between patients with and without HCC in their study of 19 patients. The effect of tumor-recurrence on HRQoL could not be evaluated in this study since tumor-recurrence was noted in only 1 patient. Further studies of larger population samples are needed to investigate the effects of HCC, tumor recurrence and development of malignancies on HRQoL.

The present study has several limitations related mainly to the study design. First, the data were assessed by cross-sectional analysis, which is less informative than those assessed by longitudinal study. Nevertheless, the sample size was relatively large. Second, selection bias cannot be ruled out; the HRQoL data did not include those patients who were too ill to respond to the questionnaire. Third, some patients might have overestimated or underestimated their activities or may have misinterpreted the questions in the self-administered questionnaire.

## Conclusions

The HRQoL for fatigue, pain, dyspnea, insomnia and financial difficulties was clinically worse in participants of this study (patients who underwent LTx) compared to the German public. Change of the allocation mode was affected by worst physical functioning. HCC had no effect on HRQoL. However, the number of HCC patients was relatively small and this point needs to be evaluated in future studies with a larger number of HCC patients. Less educated patients, divorced patients, a longer distance to transplant center, shorter waiting time and longer hospitalization were associated with lower HRQoL-scores, probably reflecting a state of depression. Especially, these patients should be supervised carefully and treated with psychotherapeutic supervision. Working patients had better HRQoL-scores in functioning and symptom-scores. Furthermore, financial difficulties were an onerous parameter for young patients, patients with HU-status or less educated patients. These patients should be supported financially and rehabilitated in their employment status in society.

### Ethical considerations

The local ethics committee approved the study and each patient who participated in the study signed an informed consent form. The approval of the local ethics committee conformed to the provisions of the Declaration of Helsinki. This manuscript has not been published previously and is not under consideration for publication elsewhere. All authors approved this manuscript for publication and have contributed significantly to the reparation of the manuscript.

## References

[1] Starzl TE, Marchioro TL, Vonkaulla KN, Hermann G, Brittain RS, Waddell WR (1963). Homotransplantation of the Liver in Humans. Surg Gynecol Obstet.

[2] Organ Procurement and Transplantation Network. OPTN/SRTR Annual Report. http://srtr.transplant.hrsa.gov/annual_reports/2012/pdf/03_liver_13.pdf.

[3] Starzl TE, Koep LJ, Schroter GP, Hood J, Halgrimson CG, Porter KA (1979). The quality of life after liver transplantation. Transplant Proc.

[4] Braun F, Teren K, Wilms P, Gunther R, Allmann J, Broering DC (2009). Quality of life after liver transplantation. Transplant Proc.

[5] Thiel C, Landgrebe K, Knubben E, Nadalin S, Ladurner R, Grasshoff C (2013). Contributors to individual quality of life after liver transplantation. Eur J Clin Invest.

[6] Butt Z, Parikh ND, Skaro AI, Ladner D, Cella D (2012). Quality of life, risk assessment, and safety research in liver transplantation: new frontiers in health services and outcomes research. Curr Opin Organ Transplant.

[7] Elliott C, Frith J, Pairman J, Jones DE, Newton JL (2011). Reduction in functional ability is significant postliver transplantation compared with matched liver disease and community dwelling controls. Transpl Int.

[8] Saab S, Bownik H, Ayoub N, Younossi Z, Durazo F, Han S (2011). Differences in health-related quality of life scores after orthotopic liver transplantation with respect to selected socioeconomic factors. Liver Transpl.

[9] Ruppert K, Kuo S, DiMartini A, Balan V (2010). In a 12-year study, sustainability of quality of life benefits after liver transplantation varies with pretransplantation diagnosis. Gastroenterology.

[10] Bownik H, Saab S (2010). The effects of hepatitis C recurrence on health-related quality of life in liver transplant recipients. Liver Int.

[11] Castaldo ET, Feurer ID, Russell RT, Pinson CW (2009). Correlation of health-related quality of life after liver transplant with the Model for End-Stage Liver Disease score. Arch Surg.

[12] Aaronson NK, Ahmedzai S, Bergman B, Bullinger M, Cull A, Duez NJ (1993). The European Organization for Research and Treatment of Cancer QLQ-C30: a quality-of-life instrument for use in international clinical trials in oncology. J Natl Cancer Inst.

[13] Schwarz R, Hinz A (2001). Reference data for the quality of life questionnaire EORTC QLQ-C30 in the general German population. Eur J Cancer.

[14] Aberg F, Rissanen AM, Sintonen H, Roine RP, Hockerstedt K, Isoniemi H (2009). Health-related quality of life and employment status of liver transplant patients. Liver Transpl.

[15] Desai R, Jamieson NV, Gimson AE, Watson CJ, Gibbs P, Bradley JA (2008). Quality of life up to 30 years following liver transplantation. Liver Transpl.

[16] Chen PX, Yan LN, Wang WT (2012). Health-related quality of life of 256 recipients after liver transplantation. World J Gastroenterol.

[17] Huda A, Newcomer R, Harrington C, Blegen MG, Keeffe EB (2012). High rate of unemployment after liver transplantation: analysis of the United Network for Organ Sharing database. Liver Transpl.

[18] Wang GS, Yang Y, Li H, Jiang N, Fu BS, Jin H (2012). Health-related quality of life after liver transplantation: the experience from a single Chinese center. Hepatobiliary Pancreat Dis Int.

[19] Schrem H, Reichert B, Fruhauf N, Becker T, Lehner F, Kleine M (2012). The Donor-Risk-Index, ECD-Score and D-MELD-Score all fail to predict short-term outcome after liver transplantation with acceptable sensitivity and specificity. Ann Transplant.

[20] Tanikella R, Kawut SM, Brown RS, Krowka MJ, Reinen J, Dinasarapu CR (2010). Health-related quality of life and survival in liver transplant candidates. Liver Transpl.

[21] Rodrigue JR, Nelson DR, Reed AI, Hanto DW, Curry MP (2011). Is Model for End-Stage Liver Disease score associated with quality of life after liver transplantation?. Prog Transplant.

[22] Saab S, Ibrahim AB, Shpaner A, Younossi ZM, Lee C, Durazo F (2005). MELD fails to measure quality of life in liver transplant candidates. Liver Transpl.

[23] Mabrouk M, Esmat G, Yosry A, El-Serafy M, Doss W, Zayed N (2012). Health-related quality of life in Egyptian patients after liver transplantation. Ann Hepatol.

